# Recipient Reaction and Composition of Autologous Sural Nerve Tissue Grafts into the Human Brain

**DOI:** 10.3390/jcm12196121

**Published:** 2023-09-22

**Authors:** Isaac Colvett, Anah Gilmore, Samuel Guzman, Aurélie Ledreux, Jorge E. Quintero, Dhanunjaya Rao Ginjupally, Julie A. Gurwell, John T. Slevin, Zain Guduru, Greg A. Gerhardt, Craig G. van Horne, Ann-Charlotte Granholm

**Affiliations:** 1 Department of Neurosurgery, University of Colorado Anschutz Medical Campus, Aurora, CO 80045, USA; isaac.colvett@cuanschutz.edu (I.C.); anah.gilmore@cuanschutz.edu (A.G.); aurelie.ledreux@cuanschutz.edu (A.L.); 2 Department of Pathology, University of Colorado Anschutz Medical Campus, Aurora, CO 80045, USA; samuel.j.guzman@cuanschutz.edu; 3 Brain Restoration Center, University of Kentucky, Lexington, KY 40536, USA; george.quintero@uky.edu (J.E.Q.); jagur@email.uky.edu (J.A.G.); jslevin@uky.edu (J.T.S.); gregg@uky.edu (G.A.G.); craigvanhorne@uky.edu (C.G.v.H.); 4 Department of Neurosurgery, University of Kentucky, Lexington, KY 40536, USA; drdhanurao@gmail.com; 5 Department of Neuroscience, University of Kentucky, Lexington, KY 40536, USA; 6 Department of Neurosurgery, Krishna Institute of Medical Sciences, Secunderabad 500003, Telangana, India; 7 Department of Neurology, University of Kentucky, Lexington, KY 40536, USA; zain.guduru@uky.edu

**Keywords:** Parkinson’s disease, neurodegenerative disorders, nucleus basalis of Meynert, substantia nigra

## Abstract

Parkinson’s disease (PD) is a severe neurological disease for which there is no effective treatment or cure, and therefore it remains an unmet need in medicine. We present data from four participants who received autologous transplantation of small pieces of sural nerve tissue into either the basal forebrain containing the nucleus basalis of Meynert (NBM) or the midbrain substantia nigra (SN). The grafts did not exhibit significant cell death or severe host-tissue reaction up to 55 months post-grafting and contained peripheral cells. Dopaminergic neurites showed active growth in the graft area and into the graft in the SN graft, and cholinergic neurites were abundant near the graft in the NBM. These results provide a histological basis for changes in clinical features after autologous peripheral nerve tissue grafting into the NBM or SN in PD.

## 1. Introduction

Parkinson’s disease (PD) is the second most common age-related neurological disorder and affects more than 1,000,000 individuals in the USA. The clinical manifestations include tremor in hands, arms, legs, jaw, or head; altered gait; disturbance of balance and muscle coordination; and non-motor symptoms, such as cognitive impairment and sleep disturbances [1,2,3,4]. Progressive cell loss in the substantia nigra (SN) and the regression of dopaminergic (DA) terminals, beginning predominately in the posterior putamen, are histopathological hallmarks of the motor symptoms of PD [5]. Meanwhile, neurocognitive deficits in PD are associated with the loss of cholinergic fibers and neuronal phenotype in the nucleus basalis of Meynert (NBM) of the basal forebrain [6,7]. To date, no disease-modifying therapy exists to slow, alter, or reverse the progression of PD [1,2], even though dopaminergic agents [8] and surgical interventions, such as deep brain stimulation (DBS), can, for a time, minimize the symptoms [9]. There is therefore still a great need for interventions that can slow the progressive cell loss occurring in PD.

A few decades ago, transplantation studies using human fetal ventral mesencephalic tissue provided strong evidence that grafted DA neurons could reinnervate the striatum, leading to improvement in motor functions in patients with PD and in animal models [10,11,12,13,14]. Others have tried a different strategy of providing trophic support to degenerating neurons, including the infusion of glial cell line-derived neurotrophic factor (GDNF) in patients with PD [15,16,17,18] and manufacturing and temporarily implanting nerve growth factor (NGF)-producing cells targeting the NBM in patients with Alzheimer’s disease (AD) [19,20]. It is possible that a continuous delivery of cell survival factors into the grafted area could overcome pathological processes in SN and NBM neurons and therefore halt the progression of pathology. In keeping with this notion, our group published data showing that a fetal kidney co-graft, transplanted together with fetal DA neurons, in rats could substantially increase graft survival and fiber outgrowth into the recipient striatum [21]. The premise that peripheral tissues can support CNS neuronal survival has also been supported by others [10,22]. The conclusions from previous studies were that peripheral tissue could survive grafting to the brain and, furthermore, provide a source of continuous secretion of numerous much needed growth factors to the fetal co-graft.

More recent studies support the idea that peripheral nerve tissue grafts, containing fibroblasts, Schwann cells, and mesenchymal stem cells, can also provide a powerful continuous secretion of a cocktail of anti-inflammatory, pro-regenerative, neuroprotective, and growth factors needed for neuronal survival [10,22,23]. However, the host response, including activation of innate immune cells in the brain, can be detrimental to allogeneic transplants; for example, of stem cells [24,25,26,27]. This is the major reason for the investigational treatment paradigm of autologous regenerative peripheral nerve tissue (PNT) transplantation we have developed [28,29]. The peripheral nervous system can readily repair itself after injury, with a key element in this process being the re-differentiation of Schwann cells and likely other cells in the tissues into repair cells capable of releasing a variety of growth and cell survival factors [30,31].

As part of an exploratory phase I clinical trial, we examined the safety, feasibility, and tolerability of grafting autologous, injury-activated PNT—specifically, sural nerve tissue—to provide growth- and cell survival-promoting factors to the SN [28] or NBM. As part of a follow-up trial [29] included on clinicaltrials.gov (NCT02369003), participants with PD received PNT grafts at the same time as they were undergoing DBS surgery, which is an FDA-approved treatment and standard of care for treatment of advanced PD motor symptoms. The PNT was not treated or labeled to make subsequent identification possible; therefore, to date, we have not been able to verify the deposition at the target or the long-term viability of the tissue in participants. Here, we report the clinical outcomes and histopathological results from four subjects who participated in the clinical trial and who died from unrelated complications 32–55 months after receiving a unilateral PNT transplantation into the SN or the NBM in the basal forebrain. All participants received bilateral placements of DBS leading into the globus pallidus interna (GPi) and simultaneously a unilateral PNT transplantation. This manuscript is focused on the survival of grafted cells, as well as innervation from the host basal forebrain or SN neurons.

## 2. Materials and Methods

***Clinical trial background*:** The participants described here were 4 of the 67 participants who participated in the clinical trial (NCT02369003) that has been described, in part, in [29]. The inclusion criteria for the trial were: (1) undergoing DBS of the subthalamic nucleus or GPi, (2) age range 40–75, (3) able to give informed consent, (4) positive response to carbidopa-levodopa, (5) no significant cognitive deficit per a formal neuropsychological exam, and (6) able to tolerate the surgical procedure. The exclusion criteria included: (1) previous PD surgery or intracranial surgery; and (2) females who were pregnant, lactating, or of child-bearing potential and unwilling to use an adequate birth control method during the period of the study. Participants were allocated to receive PNT transplantation to the SN or NBM, in part, based on the order in which they were enrolled and the absence or presence of cognitive deficits/mild cognitive impairment following a formal neuropsychological exam.

***Dissection of brain tissue*:** The study was approved by the University of Kentucky’s Institutional Review Board and participants provided informed consent and enrolled in the phase I trial at clinicaltrials.gov (NCT02369003). *PNT implantation:* As described in [29], during the time of DBS surgery, after the sural nerve was identified for each participant, a biopsy was taken, and the fascicles were removed from the nerve to resize and reshape them for delivery using a standard guide tube with a side window for loading of the tissue. The guide tube had dimensions of 5 mm in length and 1.6 mm in diameter. The implanted PNT was composed of about five segments, each measuring approximately 1 mm. These segments were obtained from fascicles that were harvested and dissected from the distal portion of the sural nerve. This nerve had been transected as part of the initial phase of the multi-stage DBS surgery. All participants reported here received DBS electrodes targeting the GPi. The target selection was a clinical decision based on routine assessments performed as part of the standard of care at the University of Kentucky. The target was identified using direct targeting of the GPi utilizing MRI scans and microelectrode recordings. PNT was implanted, contralateral to the most affected side based on lateralized scores of a practically defined off-state Unified Parkinson’s Disease Rating Scale (UPDRS) Part III exam, to unilaterally target the SN or NBM based on direct imaging of trajectories (Elements, Brainlab, Munich, Germany).

For each participant, *postmortem* consent was obtained from the next of kin, and the brain was rapidly removed. The two hemispheres were fixed in 4% paraformaldehyde for two weeks and then sliced in 1 cm coronal slices and subjected to additional fixation free-floating in 4% paraformaldehyde in PB for one more week. The brain tissue was then transferred to a 30% sucrose in phosphate buffer (PB), followed by transfer to a cryoprotection solution in PB (30% glycerol, 30% ethylene glycol, and 40% PO4 buffer), and then stored at −20 °C until dissection. Due to technical difficulties at autopsy, it was not possible to generate free-floating SN or putamen sections for unbiased stereological assessments of cell numbers for PD1. Anatomical blocks from 16 brain regions were dissected from the 1 cm slabs, embedded in paraffin, and cut at a thickness of 5 µm on a Microm microtome (Thermo Fisher Scientific Inc., Waltham, MA, USA). For the SN and NBM regions, 2 mm thick sections were generated from each side and embedded in paraffin separately. This led to three slices for each side, which were named SN or NBM1, 2, and 3, respectively, for each hemisphere, with SN/NBM1 separated from SN/NBM3 by approximately 6 mm in the coronal plane. Five-micron sections were then generated, and every 12th section was stained with hematoxylin and eosin (H&E) and antibodies directed against tyrosine hydroxylase (TH) for identification of DA cell bodies and neurites. For the caudate and putamen, thicker blocks were generated for the anterior vs. posterior portions of each region. For these, every 36th section was stained with TH antibodies to determine caudate/putamen innervation of each hemisphere separately.

***Immunohistochemistry:*** Paraffin-embedded sections were deparaffinized and pre-treated for antigen retrieval. For neuropathological staging, Bielschowsky silver staining and hematoxylin and eosin routine staining, as well as immunohistochemical staining, were performed in cortical and subcortical brain regions using primary antibodies directed against tyrosine hydroxylase (TH) (cat: AB113, Abcam Inc., Cambridge, MA, USA, 1:100), alpha-synuclein (cat: AB5038, Millipore, Darmstadt, Germany, 1:250), p75 neurotrophin receptors (p75NTR, cat: MAB367 R&D Systems, Wiesbaden, Germany, 1:250), amyloid beta 1-42 (Millipore, cat: AB5078P, dilution 1:100), and AT8 (cat: MN1020, Thermofisher, Waltham, MA, USA, 1:250). Endogenous peroxidase activity was blocked by a 7:2:1 ratio of TBS, MeOH, and 3% hydrogen peroxide (H_2_O_2_), respectively, followed by incubation with 100 mM sodium metaperiodate. Sections were blocked with TBS containing 0.25% Triton-X and 10% normal serum for 1 h and then incubated overnight with the primary antibody. After washing, sections were incubated with biotin-conjugated secondary antibodies, washed in PBS, incubated with streptavidin-horseradish peroxidase (HRP) complex, visualized using a 1 mg/mL 3′,3′-diaminobenzidine (DAB) solution containing 0.02% H_2_O_2_, dehydrated, and cover-slipped.

***Immunofluorescence:*** Paraffin-embedded sections were deparaffinized and pre-treated for antigen retrieval. Staining was performed in the striatum, SN, and NBM. A list of the fluorescent stains completed on different brain areas can be found in Table 1. All immunofluorescent stains were performed at a primary antibody dilution of 1:250. Sections were blocked and permeabilized in TBS + 0.25% Triton-X, and 10% normal serum for 1 h. Autofluorescence was quenched using a TrueBlack^TM^ Lipofuscin Autofluorescence Quencher (cat: TB-250-1, Gold Biotechnology, St. Louis, MO, USA) and sections were mounted using ProLong Gold antifade reagent with DAPI (cat: P36931, Invitrogen, Carlsbad, CA, USA).

## 3. Results

### 3.1. Participant Medical History in the Study

**Figure 1:**
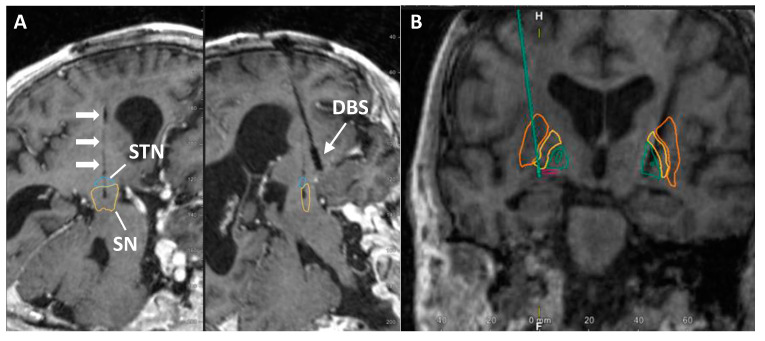
Location of graft placement in either SN (**A**) or NBM (**B**). White arrow indicates graft trajectory in **A.** Green arrow demarcates PNT graft location in **B**. For **A**: Blue—Subthalamic nucleus (STN); Yellow—SN. For **B**: Yellow—GPe; Dark green—GPi; Orange—putamen; pink—NBM.

As part of a safety, feasibility, and tolerability clinical trial, participants successfully received standard-of-care bilateral DBS with electrodes targeting the GPi and unilateral PNT implantation to either the SN or NBM (Table 2) [29]. Participants were actively followed for 2 years (Table 2) under the study protocol, except for PD2, who died from unrelated complications following a fall and pneumonia prior to completing the two-year visit. We did not observe any serious adverse events related to the study intervention in participants, and PD1, PD4, and PD5 died from non-study related events; i.e., complications related to PD: bowel obstruction, pneumonia, and pulmonary arrest, respectively. Representative MRI images of PNT placement either in the SN (A) or in the NBM (B) are shown in Figure 1 below.

### 3.2. Histology

**Figure 2:**
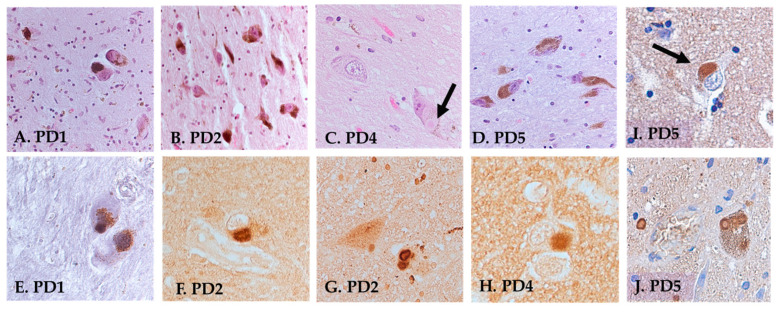
Neuropathology of PD1–5. (**A**–**D**) H&E staining of the SN in PD1, 2, 4, and 5. Note loss of neuronal cell bodies and neuromelanin, as well as LBs. (**E**–**J**) α-synuclein immunohistochemistry demonstrating LBs in various brain regions. (**E**) SN of PD1; (**F**) amygdala, PD2; (**G**) LBs in the LC of PD2; (**H**) temporal cortex LBs in PD4; (**I**) amygdala in PD5; (**J**) LC in PD5. Note cortical and subcortical LBs in all participants examined. Black arrows in C and I point to Lewy Bodies.

***Neuropathological staging*:** Staging was performed by a board-certified neuropathologist on both cortical and subcortical areas using standard H&E staining, silver staining (Bielschowsky), and antibodies directed against amyloid, p-Tau (AT8), and α-syn. A summarized description of the neuropathological findings is shown in Table 3 and representative sections are shown in Figure 2. **For PD1,** arteriosclerotic changes were observed in the SN, medulla, thalamus, amygdala, cerebellum, parietal cortex, and basal forebrain. Infrequent AT8-positive tangles were observed in the hippocampus. In cortical areas, there were arteriosclerotic changes but no obvious tangles or plaques. α-syn immunohistochemistry revealed numerous Lewy bodies (LBs) in the SN, pons, and amygdala but not in cortical areas or the thalamus. The neuropathological diagnosis for PD1 was Lewy body disease (LBD), amygdala predominant. **For PD2,** LBs were observed with α-syn immunohistochemistry in the brainstem, SN, amygdala, hippocampus, deep nuclei of the cerebellum, and parietal cortex. No amyloid plaques in any areas could be found and rare AT8-positive tangles were seen in the hippocampus. Scattered aging-related tau astrogliopathy (ARTAG) was observed with AT8 immunostaining around vessels in the amygdala and parietal cortex. The frontal cortex was negative for α-syn, as well as tangles and plaques. Sclerotic changes were observed in blood vessels in the thalamus. The neuropathological diagnosis for PD2 was LBD, neocortical (diffuse). **For PD4,** loss of pigmentation and lower numbers of locus coeruleus noradrenergic (LC-NE) neurons were observed in the pons, along with frequent LBs. SN did not show a significant loss of neurons but abundant LBs. LBs were also observed in the amygdala, parietal cortex, and deep nuclei of the cerebellum. The hippocampus exhibited loss or atrophy of neurons in the CA1 region with rare tangles. The thalamus showed mild/moderate arteriosclerosis, no tangles, and no LBs. The neuropathological diagnosis for PD4 was LBD, neocortical (diffuse). **For PD5,** silver staining revealed a few tangles but no plaques in the medial frontal gyrus. The α-syn staining revealed a few scattered LBs in the frontal cortex and more frequent and widespread LBs in the amygdala, thalamus, and occipital cortex. A denser pattern of LBs was seen in the occipital than in the frontal cortex and most frequently in the amygdala, thalamus, and brainstem. The brain was clear of amyloid staining and had only a few AT8-stained tangles in the hippocampus. The neuropathological diagnosis of PD5 was LBD, neocortical (diffuse) (see Table 3).

In accordance with the previous literature showing that PD pathology can spread to other brain regions with time [3,32,33,34,35], α-syn immunostaining strongly suggested that the clinically diagnosed PD had progressed to involve the amygdala in PD1 and, in addition, neocortical regions in PD2–5. Dementia with Lewy bodies (DLB) and Parkinson’s disease dementia (PDD) are known to share clinical and pathological hallmarks but have been considered as two separate conditions, both involving widespread LBs [3,32], and may be difficult to discern from each other using neuropathological staging. Based on clinical observations in these patients, their initial clinical diagnosis of PD had progressed, as identified by pathologic evaluation, with a spread of LBs into subcortical and cortical areas, rendering a pathologic diagnosis of LBD, neocortical (diffuse) (see Figure 2 and Table 3).

**Figure 3:**
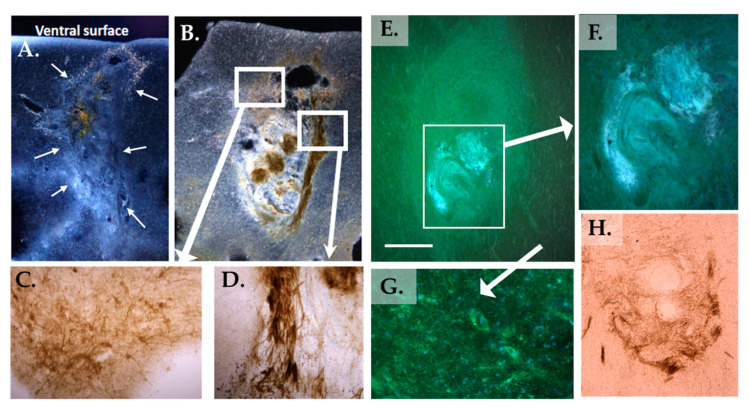
Morphology of NBM grafts. (**A**–**D**) NBM graft in PD2 stained for ChAT (**A**) and AChE (**B**–**D**). (**A**,**B**) Darkfield images. The graft was located near the ventral surface (arrows in (**A**)). (**B**,**D**) Cholinergic fiber bundles were seen inside the graft, forming fascicle-like structures. (**C**) Basal forebrain cholinergic neurons dorsal of the graft. (**E**–**H**) NBM graft in PD4. Cholinergic fibers and cell bodies were seen in a fluorescent ChAT stain (**E**–**G**), as well as in an AChE stain (**H**). As in PD2, these cholinergic fiber bundles sometimes formed fascicles. Scale bar in (**E**) represents 500 microns for (**A**,**B**,**E**).

**Figure 4:**
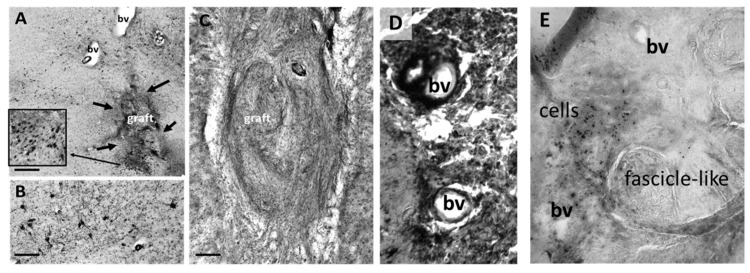
(**A**,**B**) p75 NTR immunostaining of the PD2 graft and surrounding NBM. Note a dense pattern of p75-stained cell bodies within the graft (arrows and inset in (**A**)), along with immunostained neuronal cell bodies in the overlying NBM (**B**). In (**C**), routine staining of the PD2 graft shows the distinct morphology of the graft at 29 months post-grafting, with both islands of cell bodies and streamlined tissues resembling fascicles (see also (**E**)). Scale bars represent 200 microns (**A**), 75 microns (**B**), and 100 microns (**C**). H&E staining shows numerous blood vessels (bv) in PNT grafts (**D**,**E**).

***Graft Characteristics:*** Sections with obvious grafts were found for PD2, PD4, and PD5. Due to poor tissue fixation, the graft was not readily identifiable in PD1. No abnormal growth was detected in any areas surrounding the grafts. In PD2 and 4, grafted tissues were observed near the ventral surface of the basal forebrain area (Figure 3) and displayed cholinergic fiber growth in the graft/host border, as well as inside the graft (Figure 3). Additionally, a dense pattern of P75NTR-positive cells was discovered delineating the graft, as well as inside the graft tissue (Figure 4), suggesting that the origin for the P75NTR small cell bodies may stem from the PNT [36,37]. As with PD2, cholinergic fibers were found near and inside the NBM graft in PD4 (Figure 3). This indicates cholinergic innervation of the graft by the host brain tissue, as the PNT would not be expected to express these markers.

As seen in Figure 4, blood vessels, as well as objects that closely resembled fibrous fascicles and islands with cell bodies, were observed within the grafted tissue, characteristic of peripheral nerve tissue as described by our group and others [29].

**Figure 5:**
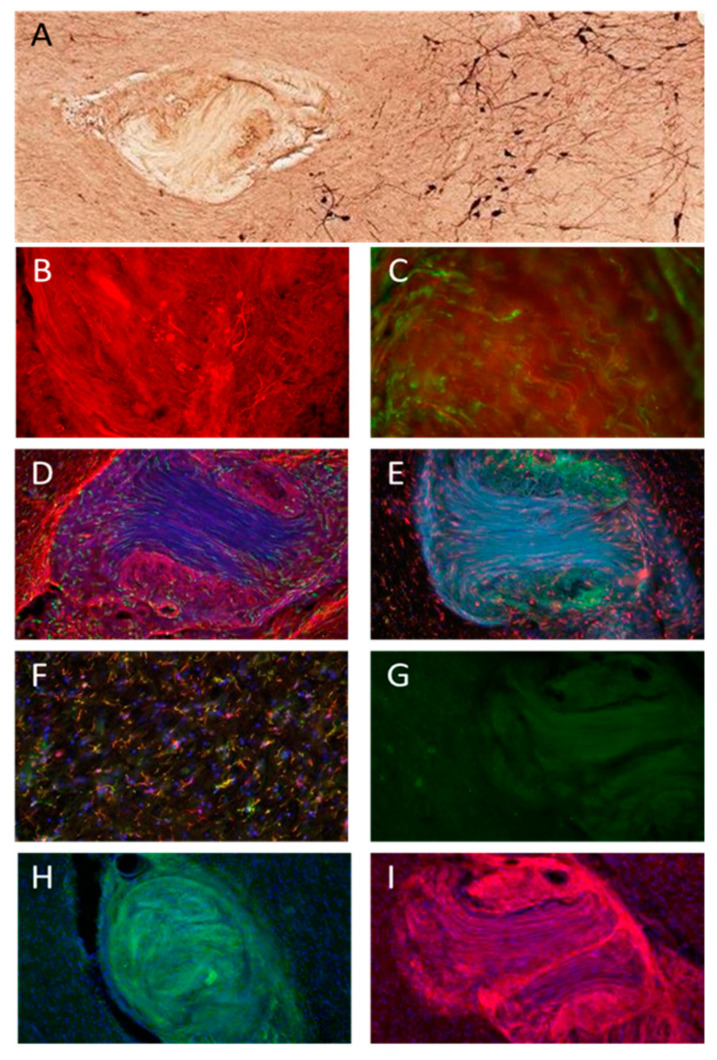
Immunostaining of the PD5 graft. TH fibers were seen inside the graft (**A**–**C**), as were Gap43-positive fibers (green areas in (**C**)). This indicates active growth of DA fibers from the SN. Staining for IBA1 and GFAP revealed IBA1-positive cells inside the graft (green areas in (**D**)), as well as a thin halo of GFAP reactivity at the host–graft interface (red areas in (**D**)). There was no colocalization of TMEM119 in the graft (red areas in (**E**)) and Iba1 (green areas in (**E**)) but extensive colocalization outside the graft (yellow areas in (**F**)). There was minimal Sox10 reactivity inside the graft (green areas in (**G**)), though there were some Sox10-positive cells outside the graft. The graft was immunoreactive for ER-TR7, a collagen marker (green areas in (**H**)), suggesting large amounts of collagen and fibroblasts inside the graft. Finally, the graft fluoresced intensely for P75NTR (red areas in (**I**)), indicating high expression of cells within the graft. Blue nuclei were stained with DAPI.

Similarly, in PD5, TH-positive fibers were seen inside the graft in the SN pars compacta (Figure 5A–C), indicating dopaminergic innervation of the grafted tissue by the host tissue. Additionally, growth-associated protein-43 (GAP-43)-positive fibers were found inside the graft (Figure 5C), further indicating new axonal growth into the graft since this protein is expressed in the growth cone of actively regenerating axons [38]. The graft in PD5 demonstrated a bright GFAP-positive border at the host–graft interface (Figure 5D), suggesting astrogliosis or glial scarring. However, this border did not extend beyond 100 microns from the graft and did not suggest an active rejection of the grafted tissue. Iba1-positive cells were also seen in the graft (Figure 5D,E). Since peripheral macrophages, as well as microglia, can express Iba1, we further stained for TMEM119, a marker that should only be present in microglia. Interestingly, TMEM119 colocalized with Iba1 outside the graft (Figure 5F) in the SN but not inside the graft (Figure 5E), suggesting the continued presence of macrophages from the grafted peripheral nerve tissue in the graft. The basic morphology of the graft in PD5 was similar to that seen in PD2 and 4, with what appeared to be fascicles of fibrous tissue surrounded by islands of cell bodies.

Sox10, a marker for Schwann cells, as well as oligodendrocytes, showed minimal reactivity inside the graft (Figure 5G). P75NTR, a nerve growth factor receptor antibody, however, showed strong positivity inside the grafted area (Figure 5I), as did ER-TR7 (Figure 5H), a marker for collagen, but neither showed prominent labeling outside of the graft. Since there are minimal fibrous proteins, such as collagen, present in the brain parenchyma [39], the findings shown in Figure 5H strongly suggest that peripheral nerve tissue survived in the graft and that fibrous collagen may have resulted from fibroblast production within the tissue. In summary, these results indicate that the grafts can express high levels of collagen and P75NTR and contain macrophages as well as host tissue axonal projections.

## 4. Discussion

These findings demonstrate innervation of the PNT autografts from surrounding cholinergic (PD2 and 4) and dopaminergic (PD5) neurons in the recipient brain. Further, three out of the four participants had a final pathologic diagnosis of Lewy body disease, neocortical (diffuse), with only the PD1 case demonstrating Lewy body disease, amygdala predominant. The pathologic diagnosis was suggestive of disease progression following the time of graft implantation. Proof of surviving peripheral cells in the graft was evident from the ER-TR7 immunostaining, which is a marker for collagen type VI and fibroblasts. Further, dense P75NTR immunoreactivity was observed within the grafted tissues both in the SN graft and the NBM grafts, and double labeling with TMEM119 and Iba1 suggested that macrophages were present in the graft but not brain-derived microglia. No major tissue rejection, reaction, or growth of the grafted tissues were observed in any of the engrafted participants, although a thin rim of astrocytes could be observed in the graft/host border in PD5 (Figure 5). Routine histology revealed that the grafts consisted of what appeared to be fascicles of collagen surrounded by areas densely populated by small cells. These findings are encouraging for future clinical studies of intracranial autografts of PNT.

Due to the lack of successful long-term disease-modifying pharmaceutical interventions for PD, there has been a renewed interest in cell-mediated strategies for disease modification of PD during the last decade [28,40,41]. We have been employing an investigational strategy to harness the regenerative capacity of the peripheral nervous system to modify the disease progression in the SN and/or NBM in PD by providing supportive and cell survival factors from grafted tissues to affected cells. Critically, because of the design of this intervention and the limitations of MR imaging for identification of grafted tissues, we had been unable to confirm (1) delivery of PNT to target and (2) whether any elements of the PNT survived delivery, much less survived for long periods of time in vivo, until we had access to *postmortem* brain tissues for this study. Previous studies in primates have shown survival of sural nerve and production of neurotrophic factors up to 3 months following grafting [22]. In the current study, we were able to ascertain the presence of P75NTR-positive cells in the graft in the implanted SN or NBM even more than 2 years post-implantation (see Figure 4 and Figure 5I), strongly suggesting the presence of grafted cells still at this time point. As can be seen in Table 2, the graft survival time ranged from 29 to 55 months post-grafting, and none of the participants examined here passed away from graft- or surgery-related complications. P75NTR can serve as an immunohistological marker for immature and non-myelinating Schwann cells in culture or in vivo [42], as well as in regenerative PNT [37], and we therefore utilized this marker to identify the presence of cells originating from the PNT. Numerous P75NTR-positive cell bodies were observed within the grafted tissues. In addition, we utilized double labeling of two markers for immune cells, Iba1 and TMEM119 [43], of which both are present in resident microglia in the brain parenchyma but TMEM119 is not present in peripheral macrophages (Figure 5E), strongly suggesting that this marker could distinguish between microglia and infiltrating macrophages in the grafted tissues. In addition, we utilized a marker for fibroblasts and collagen type VI, ER-TR7, which also stained heavily in the grafted tissue but not the surrounding brain parenchyma. ER-TR7 stains for an intracellular component of fibroblasts but does not stain for purified laminin; collagen types I, II, III, or V; or fibronectin [44], making this an excellent marker to identify peripheral nerve tissues in brain parenchyma. As shown by others [39], levels of fibrous collagen in brain parenchyma are low or nonexistent, allowing this marker to demonstrate successful survival of grafted PNT. The most prominent form of collagen in brain tissue is the non-fibrillar collagen IV, except, for example, in glioblastoma cells, which produce fibrillar proteins [45,46].

Previous work has shown that Schwann cells and fibroblasts upregulate the production of neurotrophic factors, extracellular matrix proteins, and neural cell adhesion molecule (NCAM) during neuronal survival and axonal regeneration [47,48,49,50,51,52]. Neurotrophic factors, produced by both Schwann cells and fibroblasts found in peripheral nerve tissues and which we identified in the grafts, can aid in reducing the effects of disease or injury to the brain [53], providing one potential mechanism by which PNT could be effective in increasing neuronal survival in PD or in AD. Direct evidence for GDNF and its family ligands in Iba1-positive glial cells has also been shown [54], demonstrating that several of the growth factors produced by cells within the PNT [23] could directly affect the activation of both astrocytes and microglia in the brain in a regeneration-positive manner. Future strategies to assess the efficacy of the graft on cell survival may include assessment of the viability of efferent nerve fibers from the targeted nucleus; for example, the projections from the SN to the posterior putamen following PNT engraftment.

The use of autologous PNT provided two critical advantages in advancing our ongoing clinical trials; namely, (1) the use of autologous tissue obviates the need for immunosuppressants, which can be an important consideration for this population; and (2) PNT is composed of a variety of cells containing a host of key cell survival factors [23]. The second point, that PNT contains a combination of cells, could also be viewed as a drawback, as the engrafted PNT is not enriched for a particular cell (e.g., Schwann cells [55]) or designed to produce a specific growth factor, like GDNF [56], especially with a strategy for providing neurorestorative support to cells. However, we believe that in using PNT, rather than cell suspensions, we are providing the damaged cells of the brain with a source of multi-component regenerative potential. Certainly, for those therapeutic strategies based on cell replacement of lost neurons, PNT is inadequate compared to induced pluripotent stem cell approaches designed to generate specific cells; e.g., dopaminergic cells [41].

The histological results may provide the basis for individual (open-label) improvements in the motor scores (i.e., UPDRS Part III) we reported after one year [28] that are comparable to the outcomes of a recent implantation of autologous stem cells [41]. While the lateralized component of the off-state motor exam showed lower scores (better) at 2 years from baseline, the improvements were bilateral. It is unclear why the motor exam improvements were bilateral following a unilateral implantation. Although we cannot rule out a placebo effect [57] in this participant or overperformance at motor exam assessments, bilateral effects of unilateral neurotrophic factor or cell delivery have been previously reported in animals and humans [58,59]. We note, however, that determining efficacy was beyond the scope of the primary objectives of the safety and feasibility clinical trial in which these subjects participated. Furthermore, it is unclear how to interpret the contribution of PNT to clinical outcomes from a single individual when we think the most influential factor in near-term symptom relief is through medication and DBS. The UPDRS Part III scores reported in Table 2 were obtained in the practically defined off-state when participants were off medication (at baseline) and off medication and DBS (at the two-year visit). Our overall hypothesis is that PNT might affect the survivability of cells in the areas associated with motor function (SN) or cognitive function (NBM); therefore, we would not expect that PNT delivery to the NBM would have much of an effect on motor function. Of course, future testing will have to be carried out to further evaluate this possibility.

We think further examination of this approach is merited to explore if there are consistent improvements in clinical, functional, and histological outcomes under an ongoing safety profile for this investigational intervention. The observation of P75NTR-immunoreactive cells within and in the vicinity of the graft suggests survival of PNT cells that could explain the increased TH staining in the SN or the cholinergic staining in the NBM on the ipsilateral side through significant neurotrophic actions [60,61], since fibroblasts are known to produce growth factors that can aid in regeneration of peripheral nerve grafts, at least in the periphery [50].

**Limitations:** The report from this histopathological study has several limitations. (1) This study used tissues from a small number of participants. (2) The clinical outcomes reported here were from an open-label trial and so interpretation of clinical outcomes was limited. (3) For two participants, the *postmortem* interval was over 24 h, thus possibly affecting the biochemical integrity of the tissues. (4) The fixation of one participant’s tissue was insufficient to permit quantitative analyses. Finally, (5) this was a preliminary study of brain tissue from the four participants in the study and quantitative measures were therefore lacking in terms of innervation properties and clinical evaluations of these four participants.

## 5. Conclusions

The findings described herein strongly suggest that autografts of PNT survive grafting to either the NBM or SN in the brain of participants with PD without evoking abnormal tissue responses and that the PNT can exert effects on surrounding neuronal populations and become innervated by the host brain. Further work will include quantification of surviving neuronal populations, as well as assessment of growth factors and receptors that may be secreted by the intracranial PNT autografts.

## Figures and Tables

**Table 1:** Antibodies for immunofluorescence.

Antibody	Catalog #	Company
TH	AB113	Abcam, Cambridge, UK
Gap43	PA5-34943	Invitrogen, Waltham, MA, USA
IBA1 (Gt)	NB100-1028	Novus Biologicals, Littleton, CO, USA
TMEM119	HPA051870	Sigma-Aldrich, St. Louis, MO, USA
GFAP (Gt)	NB100-53809	Novus Biologicals, Littleton, CO, USA
Sox10	AB155279	Abcam, Cambridge, UK
ER-TR7	MA1-40076	Invitrogen, Waltham, MA, USA
P75	MAB367	R&D Systems, Wiesbaden, Germany
GDNF	AF-212	Novus Biologicals, Littleton, CO, USA

**Table 2:** Demographic and surgery information.

	PD1	PD2	PD4	PD5
Age at death	75	75	70	74
Sex	M	M	M	M
Implant side	Left	Left	Right	Right
Location of implant	SN	NBM	NBM	SN
Postmortem interval (h)	9.5	4	54 *	30 *
Duration of PD diagnosis at time of surgery (years)	6	8	7	8
Time with graft (months)	33	29	55	54
BASELINE				
Off-state ^1^ UPDRS Part III	46	34	47	40
Lateralized scores (contralateral to graft/ipsilateral to graft) ^2^	17/15	17/6	17/15	18/13
TWO-YEAR VISIT				
Off-state ^1^ UPDRS Part III	37	N/A	65	35
Lateralized scores (contralateral to graft/ipsilateral to graft) ^2^	14/9	N/A	17/21	10/6

^1^ Baseline practically defined off-state: >12 h of antiparkinsonian medication, two-year visit practically defined off-state: >12 h of antiparkinsonian medication **AND** > 12 h of DBS therapy; ^2^ see [29] for details. * Delayed because of testing requirements for SARS-CoV-2.

**Table 3:** Neuropathological findings.

	PD1	PD2	PD4	PD5
Neuropathological diagnosis	LBD, amygdala predominant	LBD, neocortical	LBD, neocortical	LBD, neocortical
Cerebrovascular pathology	+++	+	+	+
Focal lesions	0	0	0	0
Tauopathy	0	+ (rare)	0	0
Amyloid deposits	0	0	0	0
α-synucleinopathy	++	++	+++	+++
Cortical α-syn	0	+	++	+
Amygdala/Thal α-syn	+	+ (not Thal)	+	+++
Glial inclusions or GCIs	0	0	0	0
Brainstem LBs	+	++	++	+++

0 = no observed profiles; + = infrequent, ++ = moderate; +++ = frequent profiles. Oligodendroglial cytoplasmic inclusions (GCIs). LBD = Lewy body disease. The neuropathological diagnosis for PD1 was LBD, amygdala predominant. All other cases were diagnosed as LBD, neocortical (diffuse).

## Data Availability

Qualified investigators may place a request for data presented here to C.G.v.H. The data are not publicly available due to confidentiality concerns.

## References

[1] Aradi S.D., Hauser R.A. (2020). Medical Management and Prevention of Motor Complications in Parkinson’s Disease. Neurotherapeutics.

[2] Espay A.J., Kalia L.V., Gan-Or Z., Williams-Gray C.H., Bedard P.L., Rowe S.M., Morgante F., Fasano A., Stecher B., Kauffman M.A. (2020). Disease modification and biomarker development in Parkinson disease: Revision or reconstruction?. Neurology.

[3] Gonzalez-Latapi P., Bayram E., Litvan I., Marras C. (2021). Cognitive Impairment in Parkinson’s Disease: Epidemiology, Clinical Profile, Protective and Risk Factors. Behav. Sci..

[4] Jia F., Fellner A., Kumar K.R. (2022). Monogenic Parkinson’s Disease: Genotype, Phenotype, Pathophysiology, and Genetic Testing. Genes.

[5] Kish S.J., Shannak K., Hornykiewicz O. (1988). Uneven Pattern of Dopamine Loss in the Striatum of Patients with Idiopathic Parkinson’s Disease. N. Engl. J. Med..

[6] Liu A.K., Chang R.C., Pearce R.K., Gentleman S.M. (2015). Nucleus basalis of Meynert revisited: Anatomy, history and differential involvement in Alzheimer’s and Parkinson’s disease. Acta Neuropathol..

[7] Horsager J., Okkels N., Hansen A.K., Damholdt M.F., Andersen K.H., Fedorova T.D., Munk O.L., Danielsen E.H., Pavese N., Brooks D.J. (2022). Mapping Cholinergic Synaptic Loss in Parkinson’s Disease: An [18F]FEOBV PET Case-Control Study. J. Park. Dis..

[8] Fox S.H., Katzenschlager R., Lim S.-Y., Barton B., de Bie R.M.A., Seppi K., Coelho M., Sampaio C. (2018). International Parkinson and movement disorder society evidence-based medicine review: Update on treatments for the motor symptoms of Parkinson's disease. Mov. Disord..

[9] Artusi C.A., Dwivedi A.K., Romagnolo A., Pal G., Kauffman M., Mata I., Patel D., Vizcarra J.A., Duker A., Marsili L. (2019). Association of Subthalamic Deep Brain Stimulation With Motor, Functional, and Pharmacologic Outcomes in Patients With Monogenic Parkinson Disease: A Systematic Review and Meta-analysis. JAMA Netw. Open..

[10] Freed W.J., Willingham G., Heim R. (1992). Effects of adrenal medulla and sciatic nerve co-grafts in rats with unilateral substantia nigra lesions. J. Neural. Transpl. Plast..

[11] Bjorklund A., Lindvall O. (2017). Replacing Dopamine Neurons in Parkinson’s Disease: How did it happen?. J. Park. Dis..

[12] Marchionini D.M., Collier T.J., Camargo M., McGuire S., Pitzer M., Sortwell C.E. (2003). Interference with anoikis-induced cell death of dopamine neurons: Implications for augmenting embryonic graft survival in a rat model of Parkinson’s disease. J. Comp. Neurol..

[13] Shinoda M., Hudson J.L., Stromberg I., Hoffer B.J., Moorhead J.W., Olson L. (1995). Allogeneic grafts of fetal dopamine neurons: Immunological reactions following active and adoptive immunizations. Brain Res..

[14] Xu P., He H., Gao Q., Zhou Y., Wu Z., Zhang X., Sun L., Hu G., Guan Q., You Z. (2022). Human midbrain dopaminergic neuronal differentiation markers predict cell therapy outcomes in a Parkinson’s disease model. J. Clin. Investig..

[15] D’Anglemont de Tassigny X., Pascual A., Lopez-Barneo J. (2015). GDNF-based therapies, GDNF-producing interneurons, and trophic support of the dopaminergic nigrostriatal pathway. Implications for Parkinson’s disease. Front. Neuroanat..

[16] Torres N., Molet J., Moro C., Mitrofanis J., Benabid A.L. (2017). Neuroprotective Surgical Strategies in Parkinson’s Disease: Role of Preclinical Data. Int. J. Mol. Sci..

[17] Bondarenko O., Saarma M. (2021). Neurotrophic Factors in Parkinson’s Disease: Clinical Trials, Open Challenges and Nanoparticle-Mediated Delivery to the Brain. Front. Cell. Neurosci..

[18] Whone A.L., Boca M., Luz M., Woolley M., Mooney L., Dharia S., Broadfoot J., Cronin D., Schroers C., Barua N.U. (2019). Extended Treatment with Glial Cell Line-Derived Neurotrophic Factor in Parkinson’s Disease. J. Park. Dis..

[19] Eyjolfsdottir H., Eriksdotter M., Linderoth B., Lind G., Juliusson B., Kusk P., Almkvist O., Andreasen N., Blennow K., Ferreira D. (2016). Targeted delivery of nerve growth factor to the cholinergic basal forebrain of Alzheimer’s disease patients: Application of a second-generation encapsulated cell biodelivery device. Alzheimer’s Res..

[20] Mitra S., Behbahani H., Eriksdotter M. (2019). Innovative Therapy for Alzheimer’s Disease-With Focus on Biodelivery of NGF. Front. Neurosci..

[21] Granholm A.C., Henry S., Herbert M.A., Eken S., Gerhardt G.A., van Horne C. (1998). Kidney cografts enhance fiber outgrowth from ventral mesencephalic grafts to the 6-OHDA-lesioned striatum, and improve behavioral recovery. Cell. Transpl..

[22] Kordower J.H., Fiandaca M.S., Notter M.F., Hansen J.T., Gash D.M. (1990). NGF-like trophic support from peripheral nerve for grafted rhesus adrenal chromaffin cells. J. Neurosurg..

[23] Chau M.J., Quintero J.E., Monje P.V., Voss S.R., Welleford A.S., Gerhardt G.A., van Horne C.G. (2022). Using a Transection Paradigm to Enhance the Repair Mechanisms of an Investigational Human Cell Therapy. Cell. Transpl..

[24] Salado-Manzano C., Perpina U., Straccia M., Molina-Ruiz F.J., Cozzi E., Rosser A.E., Canals J.M. (2020). Is the Immunological Response a Bottleneck for Cell Therapy in Neurodegenerative Diseases?. Front. Cell. Neurosci..

[25] De Chiara G., Marcocci M.E., Sgarbanti R., Civitelli L., Ripoli C., Piacentini R., Garaci E., Grassi C., Palamara A.T. (2012). Infectious agents and neurodegeneration. Mol. Neurobiol..

[26] Fainstein N., Ben-Hur T. (2018). Brain Region-Dependent Rejection of Neural Precursor Cell Transplants. Front. Mol. Neurosci..

[27] Ideguchi M., Shinoyama M., Gomi M., Hayashi H., Hashimoto N., Takahashi J. (2008). Immune or inflammatory response by the host brain suppresses neuronal differentiation of transplanted ES cell-derived neural precursor cells. J. Neurosci. Res..

[28] Van Horne C.G., Quintero J.E., Slevin J.T., Anderson-Mooney A., Gurwell J.A., Welleford A.S., Lamm J.R., Wagner R.P., Gerhardt G.A. (2018). Peripheral nerve grafts implanted into the substantia nigra in patients with Parkinson’s disease during deep brain stimulation surgery: 1-year follow-up study of safety, feasibility, and clinical outcome. J. Neurosurg..

[29] Quintero J.E., Slevin J.T., Gurwell J.A., McLouth C.J., El Khouli R., Chau M.J., Guduru Z., Gerhardt G.A., van Horne C.G. (2022). Direct delivery of an investigational cell therapy in patients with Parkinson’s disease: An interim analysis of feasibility and safety of an open-label study using DBS-Plus clinical trial design. BMJ Neurol. Open..

[30] Brushart T.M., Aspalter M., Griffin J.W., Redett R., Hameed H., Zhou C., Wright M., Vyas A., Höke A. (2013). Schwann cell phenotype is regulated by axon modality and central–peripheral location, and persists in vitro. Exp. Neurol..

[31] Jessen K.R., Arthur-Farraj P. (2019). Repair Schwann cell update: Adaptive reprogramming, EMT, and stemness in regenerating nerves. Glia.

[32] Jellinger K.A., Korczyn A.D. (2018). Are dementia with Lewy bodies and Parkinson’s disease dementia the same disease?. BMC Med..

[33] Atik A., Stewart T., Zhang J. (2016). Alpha-Synuclein as a Biomarker for Parkinson’s Disease. Brain Pathol..

[34] Henderson M.X., Trojanowski J.Q., Lee V.M. (2019). Alpha-Synuclein pathology in Parkinson’s disease and related alpha-synucleinopathies. Neurosci. Lett..

[35] Tysnes O.B., Storstein A. (2017). Epidemiology of Parkinson’s disease. J. Neural Transm..

[36] Sardella-Silva G., Mietto B.S., Ribeiro-Resende V.T. (2021). Four Seasons for Schwann Cell Biology, Revisiting Key Periods: Development, Homeostasis, Repair, and Aging. Biomolecules.

[37] Yla-Kotola T.M., Kauhanen M.S., Asko-Seljavaara S.L., Haglund C.H., Tukiainen E., Leivo I.V. (2008). P75 nerve growth factor receptor is expressed in regenerating human nerve grafts. J. Surg. Res..

[38] Okada M., Kawagoe Y., Sato Y., Nozumi M., Ishikawa Y., Tamada A., Yamazaki H., Sekino Y., Kanemura Y., Shinmyo Y. (2021). Phosphorylation of GAP-43 T172 is a molecular marker of growing axons in a wide range of mammals including primates. Mol. Brain.

[39] Sood D., Chwalek K., Stuntz E., Pouli D., Du C., Tang-Schomer M., Georgakoudi I., Black L.D., Kaplan D.L. (2016). Fetal brain extracellular matrix boosts neuronal network formation in 3D bioengineered model of cortical brain tissue. ACS Biomater. Sci. Eng..

[40] Barker R.A. (2019). Designing stem-cell-based dopamine cell replacement trials for Parkinson’s disease. Nat. Med..

[41] Schweitzer J.S., Song B., Herrington T.M., Park T.-Y., Lee N., Ko S., Jeon J., Cha Y., Kim K., Li Q. (2020). Personalized iPSC-Derived Dopamine Progenitor Cells for Parkinson’s Disease. N. Engl. J. Med..

[42] Mirsky R., Woodhoo A., Parkinson D.B., Arthur-Farraj P., Bhaskaran A., Jessen K.R. (2008). Novel signals controlling embryonic Schwann cell development, myelination and dedifferentiation. J. Peripher. Nerv. Syst..

[43] Young K.F., Gardner R., Sariana V., Whitman S.A., Bartlett M.J., Falk T., Morrison H.W. (2021). Can quantifying morphology and TMEM119 expression distinguish between microglia and infiltrating macrophages after ischemic stroke and reperfusion in male and female mice?. J. Neuroinflamm..

[44] Van Vliet E., Melis M., Foidart J.M., Van Ewijk W. (1986). Reticular fibroblasts in peripheral lymphoid organs identified by a monoclonal antibody. J. Histochem. Cytochem..

[45] Favor J., Gloeckner C.J., Janik D., Klempt M., Neuhauser-Klaus A., Pretsch W., Schmahl W., Quintanilla-Fend L. (2007). Type IV procollagen missense mutations associated with defects of the eye, vascular stability, the brain, kidney function and embryonic or postnatal viability in the mouse, Mus musculus: An extension of the Col4a1 allelic series and the identification of the first two Col4a2 mutant alleles. Genetics.

[46] Pointer K.B., Clark P.A., Schroeder A.B., Salamat M.S., Eliceiri K.W., Kuo J.S. (2017). Association of collagen architecture with glioblastoma patient survival. J. Neurosurg..

[47] Hopf A., Schaefer D.J., Kalbermatten D.F., Guzman R., Madduri S. (2020). Schwann Cell-Like Cells: Origin and Usability for Repair and Regeneration of the Peripheral and Central Nervous System. Cells.

[48] Powers C.J., McLeskey S.W., Wellstein A. (2000). Fibroblast growth factors, their receptors and signaling. Endocr. Relat. Cancer.

[49] Barton M.J., John J.S., Clarke M., Wright A., Ekberg J. (2017). The Glia Response after Peripheral Nerve Injury: A Comparison between Schwann Cells and Olfactory Ensheathing Cells and Their Uses for Neural Regenerative Therapies. Int. J. Mol. Sci..

[50] Lu Y., Li R., Zhu J., Wu Y., Li D., Dong L., Li Y., Wen X., Yu F., Zhang H. (2019). Fibroblast growth factor 21 facilitates peripheral nerve regeneration through suppressing oxidative damage and autophagic cell death. J. Cell. Mol. Med..

[51] Rhode S.C., Beier J.P., Ruhl T. (2021). Adipose tissue stem cells in peripheral nerve regeneration-In vitro and in vivo. J. Neurosci. Res..

[52] Sullivan R., Dailey T., Duncan K., Abel N., Borlongan C.V. (2016). Peripheral Nerve Injury: Stem Cell Therapy and Peripheral Nerve Transfer. Int. J. Mol. Sci..

[53] Palasz E., Wysocka A., Gasiorowska A., Chalimoniuk M., Niewiadomski W., Niewiadomska G. (2020). BDNF as a Promising Therapeutic Agent in Parkinson’s Disease. Int. J. Mol. Sci..

[54] Kotliarova A., Sidorova Y.A. (2021). Glial Cell Line-Derived Neurotrophic Factor Family Ligands, Players at the Interface of Neuroinflammation and Neuroprotection: Focus Onto the Glia. Front. Cell. Neurosci..

[55] Gant K.L., Guest J.D., Palermo A.E., Vedantam A., Jimsheleishvili G., Bunge M.B., Brooks A.E., Anderson K.D., Thomas C.K., Santamaria A.J. (2022). Phase 1 Safety Trial of Autologous Human Schwann Cell Transplantation in Chronic Spinal Cord Injury. J. Neurotrauma.

[56] Akhtar A.A., Gowing G., Kobritz N., Savinoff S.E., Garcia L., Saxon D., Cho N., Kim G., Tom C.M., Park H. (2018). Inducible Expression of GDNF in Transplanted iPSC-Derived Neural Progenitor Cells. Stem Cell Rep..

[57] Quattrone A., Barbagallo G., Cerasa A., Stoessl A.J. (2018). Neurobiology of placebo effect in Parkinson’s disease: What we have learned and where we are going. Mov. Disord..

[58] Slevin J.T., Gerhardt G.A., Smith C.D., Gash D.M., Kryscio R., Young B. (2005). Improvement of bilateral motor functions in patients with Parkinson disease through the unilateral intraputaminal infusion of glial cell line-derived neurotrophic factor. J. Neurosurg..

[59] Spencer D.D., Robbins R.J., Naftolin F., Marek K.L., Vollmer T., Leranth C., Roth R.H., Price L.H., Gjedde A., Bunney B.S. (1992). Unilateral transplantation of human fetal mesencephalic tissue into the caudate nucleus of patients with Parkinson’s disease. N. Engl. J. Med..

[60] Mansbridge J.N., Liu K., Pinney R.E., Patch R., Ratcliffe A., Naughton G.K. (1999). Growth factors secreted by fibroblasts: Role in healing diabetic foot ulcers. Diabetes Obes. Metab..

[61] Han Y., Yang J., Fang J., Zhou Y., Candi E., Wang J., Hua D., Shao C., Shi Y. (2022). The secretion profile of mesenchymal stem cells and potential applications in treating human diseases. Signal Transduct. Target. Ther..

